# Systemic Adverse Events Associated with Locally Administered Corticosteroids

**DOI:** 10.3390/children11080951

**Published:** 2024-08-06

**Authors:** Femke De Vleeschhauwer, Kristina Casteels, Ilse Hoffman, Marijke Proesmans, Anne Rochtus

**Affiliations:** 1 Department of Pediatrics, University Hospitals Leuven, 3000 Leuven, Belgium; 2 Department of Development and Regeneration, KU Leuven, 3000 Leuven, Belgium

**Keywords:** child, inhaled corticosteroids, intranasal corticosteroids, topical corticosteroids, adverse events, growth, hypothalamic-pituitary-adrenal axis

## Abstract

Topical corticosteroids are a mainstay in the treatment of many pediatric disorders. While they have proven beneficial therapeutic effects and are generally considered safe, systemic adverse events may occur. This study presents four cases of children who experienced systemic adverse events after using inhaled and intranasal topical corticosteroids, as well as topical corticosteroids in other forms. A comprehensive literature review was performed to explore the existing evidence on this topic. The aim of this study is to raise awareness among healthcare providers about the possibility of systemic adverse events associated with the use of locally administered corticosteroids in pediatric patients. This information underscores the importance of careful monitoring, individualized treatment plans, and further research to better understand and mitigate the risks associated with corticosteroids, even those not given systemically.

## 1. Introduction

The use of corticosteroids is widely accepted as a therapy for many pediatric disorders [1]. Corticosteroids exhibit potent anti-inflammatory and immunosuppressive effects, making them valuable in the treatment of various conditions. However, despite their important therapeutic benefits, concerns persist regarding potential adverse events [1,2]. Corticosteroids exert their effects through modulation of numerous pathways in the body, which can lead to a range of adverse events. In children, the use of systemic corticosteroids can lead to growth retardation, hypothalamic–pituitary–adrenal (HPA) axis suppression, Cushing syndrome, osteoporosis, hyperglycemia and obesity [3].

One of the most-discussed adverse events is an effect on growth. Corticosteroids can negatively influence growth by interfering with endocrine and metabolic pathways essential for growth, such as growth-hormone secretion, bone formation and collagen formation [4]. Even small doses of oral corticosteroids can have a growth-suppressing effect [4]. Effects on growth after the use of topical corticosteroids, such as inhaled or intranasal corticosteroids, have also been described and remain a topic of debate [5,6,7,8]. Furthermore, systemic side effects, such as suppression of the hypothalamic–pituitary–adrenal (HPA) axis, have also been reported with the use of locally administered corticosteroids [3,9,10]. This suppression is due to the negative feedback exerted by exogenous corticosteroids on the HPA axis, leading to decreased secretion of corticotropin-releasing hormone (CRH) from the hypothalamus and adrenocorticotropic hormone (ACTH) from the anterior pituitary gland, as well as decreased levels of endogenous cortisol. Prolonged exposure and high doses can lead to HPA axis deficiency, adrenal-gland atrophy and adrenal suppression. Subsequently, the production of endogenous cortisol is insufficient [9].

This article presents four cases of children who experienced systemic adverse events after using inhaled, intranasal, and other topical corticosteroids. Through a comprehensive literature review, this study aims to provide insights into the existing knowledge regarding systemic adverse events associated with different types of locally administered corticosteroids. The objective of this study is to raise awareness about the potential risks of growth retardation and other adverse events in pediatric patients who frequently use inhaled, intranasal or topical corticosteroids. In addition, various other factors that may influence the occurrence of adverse events associated with corticosteroid use will be discussed. Through optimization of the care and follow-up of children treated with corticosteroids, systemic side effects can be effectively monitored and minimized.

## 2. Materials and Methods

### 2.1. Case Series

This study reports four pediatric cases from University Hospital of Leuven (Belgium). Pediatric patients were included if they had used a locally administered corticosteroids and if impact on growth or suppression of the hypothalamic–pituitary–adrenal axis was seen. Ethical approval was obtained from Ethics Committee Research UZ Leuven (MP055518). Informed consent was signed by the parents.

### 2.2. Literature Review

The relevant literature was searched for in the medical databases PubMed, Embase and Cochrane. The following search terms were used: child, inhaled corticosteroids, intranasal corticosteroids, topical corticosteroids, targeted corticosteroids, adverse effects, adverse events and side effects. The literature published before October 2023 was analyzed, and relevant literature was selected based on the title and abstract. In addition, a search was also performed on the reference lists of the selected articles to identify other potentially relevant studies. For the selection of studies, the following inclusion criteria were used: pediatric population (age 0–18 year), locally administered corticosteroids, preferably comparison with placebo and adverse events.

## 3. Results

### 3.1. Case Series

#### 3.1.1. Case 1

**Figure 1:**
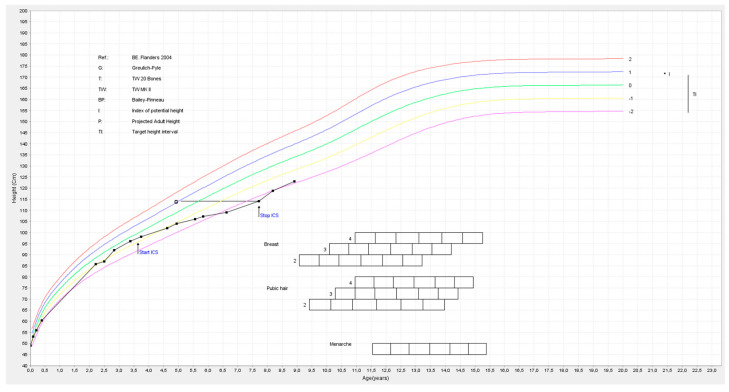
Growth chart of patient 1. Arrows indicate start and stop of corticosteroids (CS). The horizontal line refers to bone age per Greulich & Pyle. Thee vertical line refers to the target area by mid-parental height.

A 7-year-old girl was referred to a pediatric endocrinology consultation because of concerns about her linear growth. She was born at 37 weeks gestational age and had an appropriate height and weight for gestational age (respectively SD +0.25 and SD −0.4). She was briefly admitted to the neonatal intensive care unit because of respiratory distress syndrome for short-term administration of continuous positive airway pressure. The maternal history included diabetes type 1 and pre-eclampsia. From the age of three, she had been using inhaled corticosteroids for recurrent viral-induced wheezing. Initially, she was prescribed fluticasone inhalation therapy (50 mcg, one puff 2×/day), which was followed by a combination of salmeterol and fluticasone (25 mcg/50 mcg, two puffs 2×/day) as maintenance therapy. Her growth chart confirmed decreased linear growth (Figure 1), with her height standard deviation (SD) decreasing from −1 at age 2 to −2.4 at age 7. Her height velocity SD was −3.5 SD. Her general physical examination was normal and prepubertal. Her body mass index (BMI) varied between −0.5 and −0.8 SD during and after therapy. Laboratory investigations showed no abnormalities (especially no anemia or signs of HPA-axis suppression and normal kidney function, liver tests, calcium, phosphate, vitamin D, thyroid function and insulin-like growth factor-1 (IGF-1)). Her karyotype showed her to be 46,XX. Her calculated bone age SD on wrist radiographs was −2.55 (bone age was 5.1 y for her chronological age of 7 y according to Greulich and Pyle). Other factors such as poor nutrition and severe stress may also have influenced her temporarily delayed growth. Inhaled corticosteroids no longer seemed indicated at the age of 7, and following discontinuation, the patient experienced catch-up growth, with a remarkable increase in height velocity (+5.9 SD). Her height subsequently improved to −1.9 SD. As-needed therapy with a short-acting beta agonist proved sufficient for managing her respiratory complaints.

#### 3.1.2. Case 2

**Figure 2:**
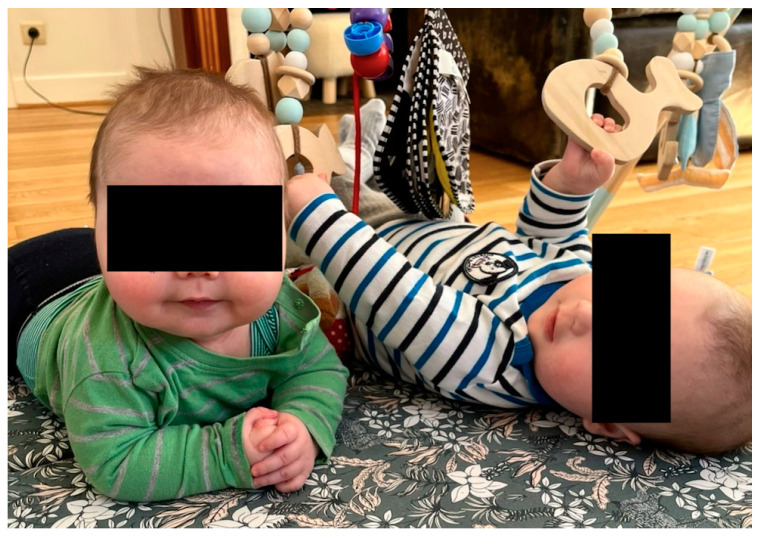
Moon facies case 2 (in comparison with his twin brother on the right side).

A 4-month-old boy presented for a follow-up consultation following a hospital admission for bronchiolitis 2 months earlier. At hospital admission, the parents reported severe and persistent nasal obstruction since birth with obstructive breathing. After excluding anatomical problems, the ENT specialist prescribed 5 days of oxymetazoline, to be followed by 6 weeks of Maxidex nasal drops (dexamethasone 1 mg/mL, 1 drop per nostril once a day). Clinical examination revealed features consistent with Cushing syndrome, including weight gain (evolution from −0.8 SD to −0.2 SD in 6 weeks), moon facies, telangiectasia, excessive hair growth and muscle weakness (Figure 2). Parents reported that the drops were difficult to administer, and they had an unclear view on the drop dispenser. Laboratory investigations revealed a tertiary adrenal insufficiency characterized by undetectable ACTH levels (<1.5 ng/L) and low cortisol levels (1.3 µg/dL in the late afternoon), which were attributed to the sudden withdrawal of exogenous steroid use. Hydrocortisone replacement was initiated and tapered gradually over 4 weeks. After 6 weeks, cortisol levels normalized (10 µg/dL in the morning), but ACTH levels were still suppressed (<1.5 ng/L). After another 3 months, ACTH levels started to increase, and the boy recovered completely.

#### 3.1.3. Case 3

An 11-year-old boy was diagnosed with eosinophilic colitis, and treatment was initiated with topically beclometasondipropionate (3 mg suppository 1×/day + 10 mg Clipper, a gastro-resistant tablet containing beclometasondipropionate, 1×/day for 4 weeks, followed by a taper to 5 mg for 4 more weeks). One month later, topical treatment with corticosteroids was stopped due to an episode of intense fear, a known side effect of Clipper. A blood test at that moment showed immeasurably low ACTH levels (<1.5 ng/L and cortisol 0.2 µg/dL in the evening), suggestive of tertiary adrenal insufficiency after topically used corticosteroids. Clinically, however, the patient did not exhibit clinical signs of Cushing. His BMI varied between −0.6 and −0.8 SD during and after therapy. The medical history revealed intermittent use of topical corticosteroids for eczema. According to the parents, the corticosteroids were always used for short durations and at minimal dosages. Following cessation of corticosteroid treatment, ACTH levels returned to normal levels after one month (31.6 ng/L), as did cortisol levels (9.9 µg/dL in the morning). Further treatment with an elimination diet for eosinophilic colitis was tried.

#### 3.1.4. Case 4

**Figure 3:**
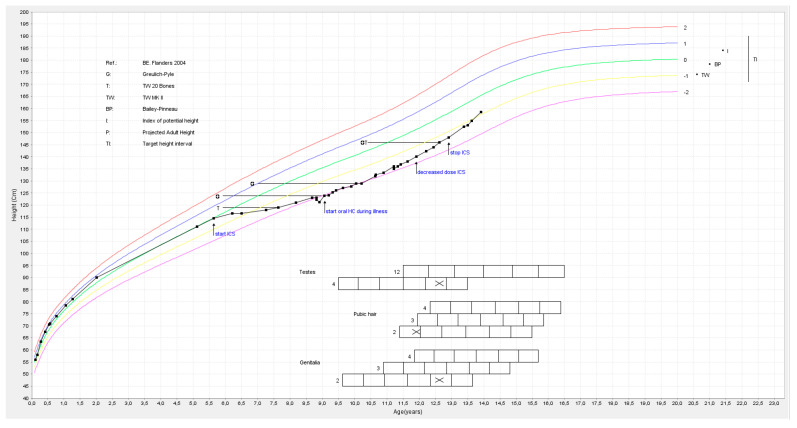
Growth chart patient 4. Arrows indicate start and stop of corticosteroids (CS). The horizontal line refers to bone age by Greulich & Pyle. The vertical line refers to the target area by midparental height. (X indicates Tanner score at the age of 12).

An almost-9-year-old boy presented to the emergency department with loss of consciousness and convulsions due to hypoglycemia of 22 mg/dL. At that time, an immeasurably low cortisol level was detected (ACTH unknown). He had a fever and had been vomiting for 1 day. IV glucose 10% and hydrocortisone were administered in the emergency department, resulting in improvement. He was referred to University Hospitals Leuven for further evaluation. His medical history included asthma, recurrent infections and various allergies. Since the age of 5.5 years, he had exhibited a declining growth curve (height SD −2.4 and height velocity SD −5.1), coinciding with intensive steroid treatment (Figure 3). Around this time, he had started using fluticasone puffs 50 mcg 2 × 2 and topical cortisone. Hydrocortisone 1% was applied over his entire body for several weeks following a generalized skin reaction to nut ingestion and skin abnormalities after he had contracted varicella. Clinically, some mild dysmorphic features were present (large thorax compared to abdomen, slight pectus excavatum, rounded facial appearance, rounded shoulder posture with slender limb build). His weight varied between −1.1 and −1.4 SD, and his BMI was always below 0 SD. His Tanner score was A1P1G1. Extensive investigations (including brain MRI, abdominal ultrasound and metabolic, immunological and genetic assessments) have so far yielded no explanation. Levels of both ACTH (6.9 ng/L) and cortisol (<0.1 µg/dL) were low. The insulin-tolerance test did not result in a rise in cortisol (maximum cortisol 0.2 µg/dL, growth hormone max 27.6 µg/L). This central adrenal insufficiency is suspected to have resulted from suppression of hypothalamic CRH secretion following extensive topical corticosteroid use and inhalation therapy with corticosteroids. A treatment plan was devised, including administration of oral hydrocortisone 10 mg twice a day (=20 mg/m^2^) during fever or stressful situations. Subsequent infections were less severe, and the patient experienced reduced fatigue. Biochemically, spontaneous improvement of the HPA axis was observed (ACTH 47 ng/L, cortisol 4.8 µg/dL in the morning). Additionally, Seretide puffs (salmeterol 25 µg/fluticasone 125 µg) could be tapered from twice daily to once daily while maintaining good asthma control. Notably, catch-up growth was observed (height velocity SD +2.7), resulting in an increase in height SD to −1.2. However, his bone age remained 3 years behind his chronological age, suggesting the potential for further catch-up growth.

### 3.2. Literature Review

#### 3.2.1. Inhaled Corticosteroids

The effects of long-term inhaled corticosteroid (ICS) use on growth in children with mild to moderate persistent asthma have been extensively studied. Three Cochrane systematic reviews have provided valuable insights into this topic [5,11,12]. The systematic review by Zhang et al. [5] involving 14 trials with 5157 children showed a statistically significant reduction in linear growth velocity of 0.48 cm/year (95% CI −0.65 to −0.30, moderate-quality evidence). In the same article, another analysis involving 15 trials with 3275 children showed a reduction in baseline height by 0.61 cm (95% CI −0.83 to−0.38, moderate-quality evidence) during the first year of treatment [5]. However, growth suppression became less pronounced after the first year of treatment and was no longer statistically significant compared with the control group. Long-term follow-up studies have shown that treatment with high doses of ICS, such as budesonide 400 mcg/day for a mean duration of 4.3 years, may lead to a reduction in adult height of 1.2 cm (95% CI −1.90 to −0.50, *p* = 0.001 [13]. A second systematic Cochrane review investigated dose–response effects and found that higher doses of ICS lead to greater reductions in growth velocity and final adult height [11]. A systematic review by Loke et al. reported similar findings, with a mean reduction of −0.48 cm/year in growth velocity after one year and evidence of a dose–response effect [14]. A more recent Cochrane review suggests that fluticasone may have less effect on growth in comparison with beclomethasone and budesonide [12]. The choice of delivery device may also influence the effect on growth because it determines the amount of drug deposited in the lungs, from where it can then be absorbed into the systemic circulation. However, evidence from these trials was too weak to make definite decisions in choice of molecule or device.

While suppression of the hypothalamic–pituitary–adrenal (HPA) axis is rarely seen after use of inhaled corticosteroids alone at a low or medium dose, chronic exposure to high doses may lead to symptomatic adrenal suppression [3,9,15]. Furthermore, the effect of ICS on bone mineral density seems negligible when low-to-moderate doses are used, but high doses and intermittent need for systematic steroids increases the risk of bone loss [16,17].

Other adverse events associated with ICS include hypertrichosis, psychological disturbances, teeth malocclusion, gastrointestinal ulceration, although evidence on these effects is limited [17]. One study noted a significant increase of the mean HbA1c value (5.44 ± 0.75%) in asthmatic children using a low dose of ICS in comparison to the control group (5.14 ± 0.41%) [18]. However, no difference in HbA1c levels was seen with a cumulative dose or increased time of usage of ICS.

Short-term use (less than 14 days) of corticosteroids for acute respiratory conditions among young children does not seem to be associated with increased adverse events compared to placebo according to the systematic review by Fernandes et al. [19].

#### 3.2.2. Intranasal Corticosteroids

Systemic complications like growth retardation and adrenal suppression after use of intranasal corticosteroids are rare but have been reported. Data regarding the exact frequency of these complications are lacking [10].

Some studies stated that administration of intranasal corticosteroids (INCS) at the recommended doses causes no clinically significant HPA axis suppression [10,20]. However, isolated case reports of iatrogenic Cushing syndrome have been reported with the use of dexamethasone or betamethasone nasal drops [21,22,23,24,25]. Normalization of cortisol typically occurred after discontinuation of INCS and tapering of doses of hydrocortisone.

Studies regarding the effect on growth have yielded conflicting results [7,8,10]. In most RCTs, no significant difference was seen in growth velocity. The overall risk of growth retardation after using INCS for a short period at low doses seems low. However, a few studies have reported a temporary decrease in growth velocity. The impact on adult height remains unclear.

Furthermore, there is uncertainty regarding ocular complications with INCS use. To date most studies showed no significant evidence linking INCS to increased intraocular pressure in children [7,10], but further research is warranted to clarify this potential risk.

#### 3.2.3. Topical Skin Corticosteroids

Dermal corticosteroids are widely prescribed for the treatment of various dermatologic disorders. Children are more prone to developing systematic adverse events due to their higher ratio of total body surface area to body weight [2]. Other factors that can influence the risk of adverse effects include high-potency corticosteroids, quantity, frequency and duration of the treatment and the site of application [26]. Examples of high-potency topical corticosteroids include clobetasol propionate cream 0.05% and betamethasone dipropionate ointment 0.05% (classes 1–2 using the World Health Organization classification of corticosteroids) [27].

In a meta-analysis by Wood Heickman et al., the incidence of HPA axis suppression was low (respectively, 2%, 3.1% and 6.6% with low-, medium- or high-potency topical corticosteroids) and reversible when pediatric patients used a short-term corticosteroids for the treatment of atopic dermatitis [27]. No clinical symptoms of adrenal insufficiency were seen among the children included in these studies. On the contrary, after long-term application of topical corticosteroids, cases have been reported of children who developed iatrogenic Cushing Syndrome [28].

Growth retardation is seen only when high levels of topical corticosteroids are systemically absorbed. When topical corticosteroids use is ceased, catch-up growth can be expected [29].

#### 3.2.4. Targeted Gastrointestinal Corticosteroids

Budesonide and beclomethasone dipropionate are known treatment options in ulcerative colitis, as they are designed to release the product topically in the colon with fewer systemic adverse events. A Cochrane review stated that budesonide does not lead to significant suppression of the HPA axis [30]. However, a study by Zhao et al. mentioned that 4 patients out of 282 receiving beclomethasone dipropionate for ulcerative colitis had decreased mean plasma cortisol and mild signs of HPA axis suppression [31].

In the treatment of eosinophilic esophagitis, dietary modifications and treatment with proton-pump inhibitors or topical glucocorticoids are treatment options. A meta-analysis of topical glucocorticoid use in children with eosinophilic colitis reported no major adverse effects. Cortisol levels were not significantly lower in patients than in the placebo group [32].

A summary of possible systemic adverse events in children using locally administered corticosteroids is provided in Table 1.

## 4. Discussion

Locally administered corticosteroids are a mainstay in the treatment of various pediatric disorders, from asthma to atopic dermatitis. While they have proven beneficial therapeutic effects and are generally considered safe, physicians must be aware of possible adverse events [2]. This article presents four case reports of children who experiences a systemic adverse event after using locally administered corticosteroids. Since this only concerns a few cases at University Hospitals Leuven, extrapolation regarding the frequency and the size of the impact of these adverse events for all children who use locally administered corticosteroids was not possible. To provide a broader perspective, a comprehensive literature review was performed to explore the existing evidence on this topic. The aim of this study is to raise awareness among healthcare providers about the possibility of systemic adverse events associated with the use of locally administered corticosteroids in pediatric patients. This information underscores the importance of careful monitoring, individualized treatment plans, and further research to better understand and mitigate the risks associated with these medications.

### 4.1. Inhaled Corticosteroids

Inhaled corticosteroids are recommended as a first-line treatment for persistent asthma in children. Their well-established benefits outweigh the potential adverse events of using inhaled corticosteroids in this condition. Side effects are considerably reduced in comparison with those associated with oral corticosteroids [17]. Evidence suggests that regular use of inhaled corticosteroids is associated with a relatively small mean reduction in growth velocity and final adult height [5,13,14]. The effects are most pronounced in the first year of treatment, and there is no cumulative effect [5]. Small but statistically significant dose–response effects on growth were also described [11,13,14]. Fluticasone seems to inhibit the child’s growth less then beclomethasone or budesonide [12].

To minimize the risk of adverse effects, it is recommended that ICS be prescribed at the lowest effective dose to maintain asthma control in children [33]. If asthma is uncontrolled in children older than 6 years, use of a long-acting β-adrenoreceptor agonist as first step up is recommended instead of an increase in the dose of corticosteroids [16]. In addition to the potential impacts of some features of the ICS used (dose, duration, molecule and inhalation device), clinicians should consider various factors that may influence the adverse effects of ICS, such as age, severity of the underlying disease, compliance, comorbidities and inadequate nutritional intake [5,34]. In addition, poor use of ICS or not taking ICS for fear of adverse events can lead to insufficient asthma control, which in turn can impair the growth of the child [12]. Clinicians also have to pay attention to the simultaneous use of other corticosteroids or growth-suppressing medication (for example, in the fourth case) [35]. It is essential to monitor the growth of children using ICS regularly (every six months), assess their diet with particular attention to sufficient intake of calcium and vitamin D and encourage physical exercise [16]. In addition, it is important to reevaluate the therapy and the combinations of medications used during follow-up.

In pediatric trials evaluating ICS, data on height and growth velocity are often lacking or incomplete. In future trials, growth should be systematically documented to better define the long-term effect of ICS on adult height [33]. Further studies are needed to compare the effects on growth associated with using different molecules, different daily doses and different inhalation devices and to determine the role of the child’s age [5].

Other side effects that have been described after use of ICS, like suppression of the hypothalamic–pituitary–adrenal axis or decreased bone mineral density, are rare at low-to-medium doses. The Canadian Society of Allergy and Clinical Immunology advises screening for adrenal suppression when children are receiving high doses (≥500 µg/day of fluticasone propionate or equivalent; ≥400 µg/day under age 12) for longer than six months. Screening is also recommended when using a medium dose in the presence of additional risk factors such as prolonged treatment, concomitant use of other nasal or topical corticosteroids and low body mass [9]. Initial screening can be done by measuring serum cortisol in the morning. When a low level is detected or when results are normal but there is a high index of suspicion, referral to a child endocrinologist is recommended. Suppression of the HPA axis can be confirmed by a low-dose ACTH-stimulation test.

### 4.2. Intranasal Corticosteroids

Intranasal corticosteroids are widely considered the first-line treatment for many rhinologic conditions, including allergic rhinitis and various subtypes of chronic rhinosinusitis [10]. While systemic adverse events like growth retardation and adrenal suppression after using intranasal corticosteroids are rare when the recommended doses are used [10], factors such as type of corticosteroid, administration technique, frequency and duration of treatment can alter the risks of developing systemic adverse events [2].

In the second case presented, a boy developed Cushing syndrome after using intranasal corticosteroids, potentially due to overdosing and the use of a strong corticosteroid. This highlights the importance of ensuring proper administration techniques and accurately assessing the need for continued treatment. When using intranasal corticosteroids, clinicians should taper the dose based on clinical assessment and assess the need for further treatment.

As previously said, the concomitant use of other corticosteroids should be assessed. To date, there are no high-quality studies of children reporting data on the cumulative effects of the combined use of different locally administered corticosteroids [10].

### 4.3. Topical Skin Corticosteroids

Topical corticosteroids are often used for the treatment of pediatric dermatologic disorders, most frequently atopic dermatitis. The risk of HPA axis suppression is low when using mild-to-moderate topical corticosteroids, especially when they are given only for a short period of time. The likelihood of adverse effects increases with higher doses, a longer duration or use in combination with other steroids [2,27,28]. To minimize the risk of adverse effects, it is recommended that clinicians use the least potent topical corticosteroids that can effectively control the flares of the disease. Maintenance with emollients remains the standard of care. The intermittent use of topical corticosteroids for more than 4 consecutive weeks should be avoided [36]. Clinicians should educate patients and caregivers on the correct method of administration and the correct amount of topical corticosteroids to use. They should provide a regimen with a taper phase for all children who require longer treatment. In addition, caution should be exercised to avoid patients using old prescriptions or treating an undiagnosed rash with topical corticosteroids without proper evaluation [26]. After discontinuation of topical corticosteroids, normalization of HPA-axis suppression is expected within 1–10 weeks [27]. Routine testing for HPA axis suppression is not recommended when topical corticosteroids are used for less than 4 weeks, as long as there are no signs or symptoms of adrenal insufficiency. Future studies are needed to determine which children are at a higher risk of systemic absorption of topical corticosteroids [27].

### 4.4. Targeted Gastrointestinal Corticosteroids

The boy in case 3 was diagnosed with eosinophilic colitis, which is considered a rare disease. Due to insufficient data, treatment options and universal guidelines for pediatric patients are controversial. The choice of therapy is individualized for each patient and based upon their symptoms. Treatment with topical corticosteroids was started, but after one month, a blood test showed immeasurably low ACTH levels, likely due to tertiary adrenal insufficiency. Caution is warranted when using (topical) corticosteroids off-label. Is there sufficient evidence for their use? Are they needed? Monitor the treated child closely and discontinue the use of corticosteroids over time if necessary.

## 5. Conclusions

Locally administered corticosteroids are widely accepted as a therapy for many pediatric disorders; examples include inhalation corticosteroids for asthma and topical corticosteroids for atopic dermatitis. While the risks of systemic adverse events, such as growth retardation and HPA axis suppression, are considerably lower in comparison to those associated with oral glucocorticosteroids, caution is still warranted. The first step is the prudent use of corticosteroids for the right indication, along with ensuring that the correct dosing, application method and treatment duration are used. Regular follow-up of children treated with corticosteroids is crucial to monitor for and minimize the occurrence of systemic adverse events. Special attention should be paid when additional corticosteroids are given, as they increase the likelihood of adverse events.

Take-home messages:

- Caution is warranted with regard to systemic adverse events when using locally administered corticosteroids, although the risk is significantly lower compared to that associated with the use of oral corticosteroids.
- Use corticosteroids judiciously and only for appropriate indications.
- Administer the lowest effective dose, for as short a time as possible, to maintain disease control.
- Ensure correct methods and dosages of administration.
- Be cautious with the simultaneous use of other corticosteroids.
- Monitor growth and check for possible adverse events every six months.
- Assess whether the child has an adequate diet, particularly ensuring sufficient intake of calcium and vitamin D.
- Encourage regular exercise.
- Provide clear information and promote transparent communication between clinicians, the child and their parents.

## Figures and Tables

**Table 1:** Summary of systemic adverse events in children.

	Growth	HPA Axis	Other
**Inhaled CS**	Dose-related suppression Baseline height: −0.61 cm/y during the first year of treatment Final height: −1.2 cm	Dose-related suppression Symptomatic with chronic exposure to high doses (≥500 μg/day of fluticasone propionate or equivalent; ≥400 μg/day <age 12)	Decreased BMD Increase in HbA1c value of 0.3% Hypertrichosis, psychological disturbances, teeth malocclusion, gastrointestinal ulceration and cataracts; scarce
**Intranasal CS**	Short periods at low doses: low risk of growth retardation Effect on final height: unknown	FDA-approved INCS, at the recommended doses: no clinically significant HPA axis suppression Dexamethasone or bethametasone nasal drops: case reports of iatrogenic Cushing Syndrome	Increased intraocular pressure in children; no significant evidence
**Topical skin CS**	Growth retardation: only with high levels	Incidence of HPA axis suppression: respectively, 2%, 3.1% and 6.6% using low-, medium, or high-potency topical CS without clinical symptoms Long-term application: case reports of iatrogenic Cushing Syndrome	
**Gastro-intestinal targeted CS**		No significant suppression of the HPA axis in comparison to placebo group	

Legend: BMD: bone mineral density; CS: corticosteroids; HPA: hypothalamic–pituitary–adrenal; INCS: intranasal corticosteroids.

## Data Availability

The data that support the findings of this study are available on request from the corresponding author.
